# A Terahertz Identification Method for Internal Interface Structures of Polymers Based on the Long Short-Term Memory Classification Network

**DOI:** 10.3390/polym14132611

**Published:** 2022-06-27

**Authors:** Shushan Wang, Hongwei Mei, Jianjun Liu, Dabing Chen, Liming Wang

**Affiliations:** 1 Shenzhen International Graduate School, Tsinghua University, Shenzhen 518055, China; wss21@mails.tsinghua.edu.cn (S.W.); wanglm@sz.tsinghua.edu.cn (L.W.); 2 State Grid Jiangsu Electric Power Company Electric Power Research Institute, Nanjing 211103, China; 15105168885@163.com (J.L.); dabingchen@163.com (D.C.)

**Keywords:** terahertz detection, long short-term memory, interface structures, 3D imaging

## Abstract

Polymers are used widely in the power system as insulating materials and are essential to the power grid’s security and stability. However, various insulation defects may occur in the polymer., which can lead to severe insulation accidents. Terahertz (THz) detection is a novel non-destructive testing (NDT) method that is able to detect the interface structures inside polymers. The large quantity of information in the THz waveform has potential for the identification of interface types, and the long short-term memory (LSTM) network is one of the most popular artificial intelligence methods for time series data like THz waveform. In this paper, the LSTM classification network was used to identify the internal interfaces of the polymer with the reflected THz pulses of the internal interfaces. The experiment verified that it is feasible to identify and image the void interfaces and impurity interfaces in the polymer using the proposed method.

## 1. Introduction

As the scale of the power grid continues to grow, more and more insulation equipment is being used in the power grid [1]. However, during manufacturing, transportation, installation, and operation, many defects caused by human or environmental factors may occur in the insulating material [2,3,4]. These defects can cause continuous degradation of the insulation performance and eventually lead to insulation accidents, resulting in economic losses and human casualties [5,6]. Therefore, in practical applications, there is an urgent need for effective non-destructive testing (NDT) methods for internal defects.

The commonly used NDT methods have their limitations. For example, ultrasonic testing requires a coupling agent for contact detection and has high demands on the ability of operators [7]. Infrared detection can only be applied for detecting the defects that cause external temperature changes and is highly influenced by environmental conditions [8]. Microwave inspection is also a contacting method with very few feature parameters, and can only qualitatively determine the presence of defects [9]. X-ray is harmful to humans, and its strong penetration ability makes it easy to directly pass through the defect, resulting in missed detection [10].

Terahertz (THz) technology is an emerging NDT method, in which the electromagnetic waves in the THz band (0.1–10 THz) are used to achieve a non-contact, safe, and efficient NDT. It has been widely used in fields such as security screening [11,12,13], biomedicine [14,15,16], and aerospace [17,18]. Meanwhile, using the property that THz waves can detect the internal interface structures of polymers, it has also been applied in the detection of power insulation defects [19,20,21]. However, most of the existing studies focus on detecting the location, size, and shape of the internal interface using macroscopic features of THz waveform (such as peak value, time delay, and other parameters). Very few studies focus on using the microscopic features of pulses in THz waves to identify the type of the interface, which is also very meaningful for the detection. The long short-term memory (LSTM) network is one of the most common current artificial intelligence methods for processing time-series data [22,23]. It has been proven to be suitable for the THz time domain waveform data [24]. Therefore, the LSTM network has great potential for interface identification in polymers.

In this paper, an internal interface identification method is proposed for polymer detection, which is based on THz waves and LSTM classification networks. Three different artificial interface samples were made to collect THz waveform data to train the LSTM. Then, the trained LSTM was used to identify and image the internal interfaces in other samples to test its performance.

The rest of the paper is organized as follows: Section 2 presents the theoretical background of THz detection and LSTM networks. Section 3 describes the experimental system and samples. The experimental results and discussions are reported in Section 4. Section 5 is the conclusion.

## 2. Theory and Methodology

### 2.1. Terahertz Detection Theory

Assuming that a THz wave is incident vertically from medium 1 to medium 2, the refraction coefficient and the reflection coefficient at the interface “1–2” can be expressed as [25]

(1) $$t_{12}=\frac{2{\tilde{n}}_{1}\left(\omega \right)}{{\tilde{n}}_{1}\left(\omega \right)+{\tilde{n}}_{2}\left(\omega \right)}$$

(2) $$r_{21}=\frac{{\tilde{n}}_{2}\left(\omega \right)-{\tilde{n}}_{1}\left(\omega \right)}{{\tilde{n}}_{1}\left(\omega \right)+{\tilde{n}}_{2}\left(\omega \right)}$$

where ${\tilde{n}}_{1}\left(\omega \right)$ and ${\tilde{n}}_{2}\left(\omega \right)$ represent complex refractive indices of the medium 1 and medium 2, respectively.

**Figure 1:**
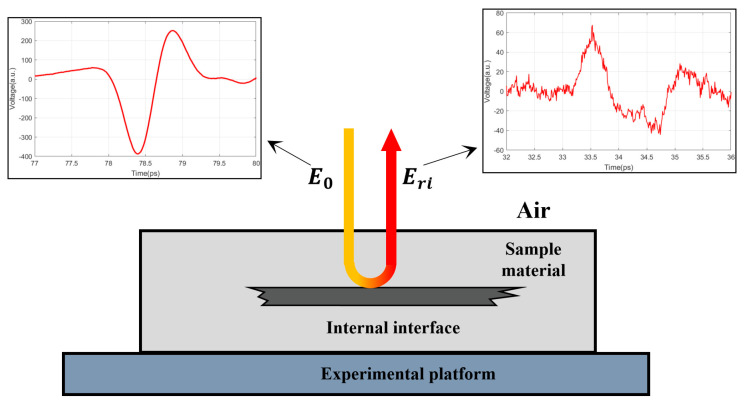
Schematic diagram of detecting an internal interface structure with THz waves.

Many insulating materials are polymers that are translucent to THz waves [26]. Thus, THz waves can propagate through insulating materials and be reflected by internal interfaces such as the defect structure. Figure 1 shows this process. A THz pulse $E_{0}$ is incident into the sample, when it encounters an internal interface structure, a reflected pulse $E_{ri}$ will be generated, which can be expressed as follows [27]:

(3) $$\;E_{ri}=E_{0}\cdot p_{a}\left(\omega ,d_{a}\right)\cdot p_{b}\left(\omega ,d_{b}\right)\cdot t_{ab}\cdot r_{bc}\cdot t_{ba}$$

where ω is the wave frequency. *a*, *b,* and *c* represent the air, sample material, and the internal interface, respectively. $d_{a}$ and $d_{b}$ are the propagating distances of THz wave in the air and the sample material, respectively. The propagation coefficient p represents the amplitude attenuation and phase change of the THz wave when propagating in the medium, which can be described as follows [27]

(4) $$p\left(\omega ,d\right)=\exp\left(\frac{-i\tilde{n}\left(\omega \right)\omega d}{c}\right)$$

where c is the vacuum speed of light.

According to Equations (1)–(4), the reflected wave $E_{ri}$ contains information about the properties of the interface structure. In turn, it is theoretically feasible to identify the interface type by the reflected wave.

### 2.2. LSTM Theory

**Figure 2:**
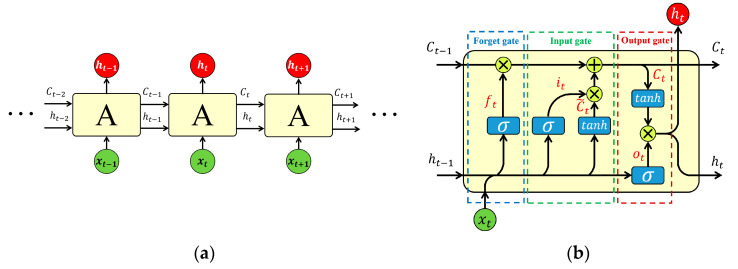
Schematic diagram of the LSTM structure. (**a**) Chain structure of LSTM and (**b**) LSTM unit architecture.

LSTM is a model structure of recurrent neural networks (RNN). In contrast with the conventional RNN, three gates are placed in each LSTM unit, including an input gate, an output gate, and a forget gate. Based on this special structure, LSTM effectively overcomes the vanishing gradient problem, which greatly limits classic RNNs [28]. A typical LSTM structure is shown in Figure 2.

As shown in Figure 2b, the forget gate output $f_{t}$ can be expressed as

(5) $$f_{t}=\sigma \left(W_{f}\cdot \left[h_{t-1},x_{t}\right]+b_{f}\right),$$

where $W_{f}$ represents the weight matrix of the forget gate, which is to be changed during training, $h_{t-1}$ is the output vector of the last unit, $x_{t}$ is the input vector of the current unit, $b_{f}$ is the bias vector, which will also be changed in the training process, and σ represents the sigmoid function, which can be expressed as

(6) $$\sigma \left(x\right)=\frac{1}{1+e^{-x}}.$$

From Equation (6), it can be seen that each number in $f_{t}$ is between 0 and 1, which represents the degree of forgetting for this part of information. Therefore, the forget gate determines which part of the information to be kept and which to be rid of.

The input gate determines which information will be used to update the cell state vector $C_{t}$. The input gate equations are:

(7) $$i_{t}=\sigma \left(W_{i}\cdot \left[h_{t-1},x_{t}\right]+b_{i}\right),$$

(8) $${\tilde{C}}_{t}=\tanh\left(W_{c}\cdot \left[h_{t-1},x_{t}\right]+b_{c}\right),$$

(9) $$C_{t}=f_{t}\;⨀\;C_{t-1}+i_{t}\;⨀\;{\tilde{C}}_{t},$$

(10) $$\tanh\left(x\right)=\frac{e^{x}-e^{-x}}{e^{x}+e^{-x}},$$

where $W_{i}$ and $W_{C}$ are the weight matrices of input gate and cell state, respectively. $b_{i}$ and $b_{c}$ are the biases of the input gate and cell state, respectively. $i_{t}$ is the output vector of the input gate, ${\tilde{C}}_{t}$ is the cell input activation vector, $C_{t}$ is the cell state vector, and ⨀ represents the element-wise product.

Finally, the output gate determines what information will be output by the following equations:

(11) $$o_{t}=\sigma \left(W_{o}\cdot \left[h_{t-1},x_{t}\right]+b_{o}\right),$$

(12) $$h_{t}=o_{t}\;⨀\;\tanh\left(C_{t}\right),$$

where $o_{t}$ is the output vector of the output gate, $h_{t}$ is the output vector of the LSTM unit, and $W_{o}$ and $b_{o}$ are the weight matrix and bias of output gate, respectively.

## 3. Experiment Setup

### 3.1. Experimental Samples

**Figure 3:**
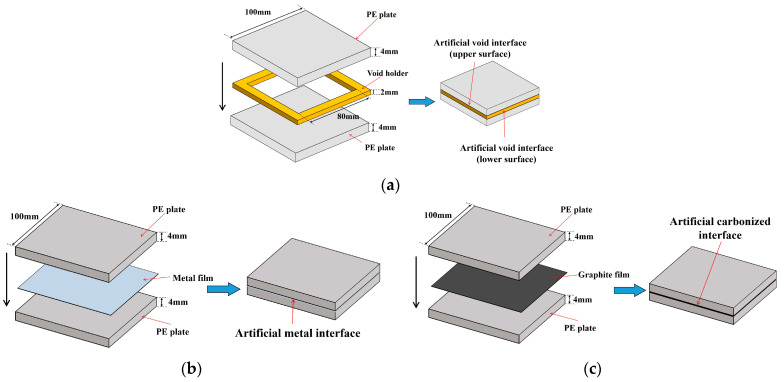
Structure of artificial interface samples for collecting LSTM training data: (**a**) artificial void interfaces; (**b**) artificial metal interface; (**c**) artificial carbonized interface.

Various interfaces can be inside insulating polymers. Void interfaces are very common in power insulation equipment, which can lead to a severe degradation of insulation performance [29]. When internal defects have developed to a certain degree, partial breakdown may occur, which can result in carbonization channels [30,31]. In addition, metal interfaces can also occur in insulation equipment, such as the metal sheaths of high-voltage cables [32], as well as metal impurities that intrude into the material during manufacture and operation [33,34]. Therefore, in this paper, three artificial interface samples were made to simulate the internal interfaces of the polymers, including the void interface, the carbonized interface, and the metal interface. Polyethylene (PE) was used as the insulating polymer. The artificial interface samples used to collect training data for LSTM are shown in Figure 3.

As shown in Figure 3, the size of the PE plate was 100 mm × 100 mm × 4 mm. For the artificial void interface sample, a resin holder with a size of 100 mm × 100 mm × 2 mm was placed between the upper and lower PE plates, which had a void area of 80 mm × 80 mm in its center. They were stacked on top of each other to simulate the upper surface (“polymer−air” interface (PA)) and the lower surface (“air−polymer” interface (AP)) of the void. To simulate metal and carbonized interfaces in the polymer, an aluminum film and a graphite film were placed between two PE plates, respectively. Different from the void interface, THz waves cannot penetrate metal and carbonized interfaces. Thus, these two artificial samples are only with the “polymer−metal” (PM) interface and the “polymer−carbonization” (PC) interface, respectively.

**Figure 4:**
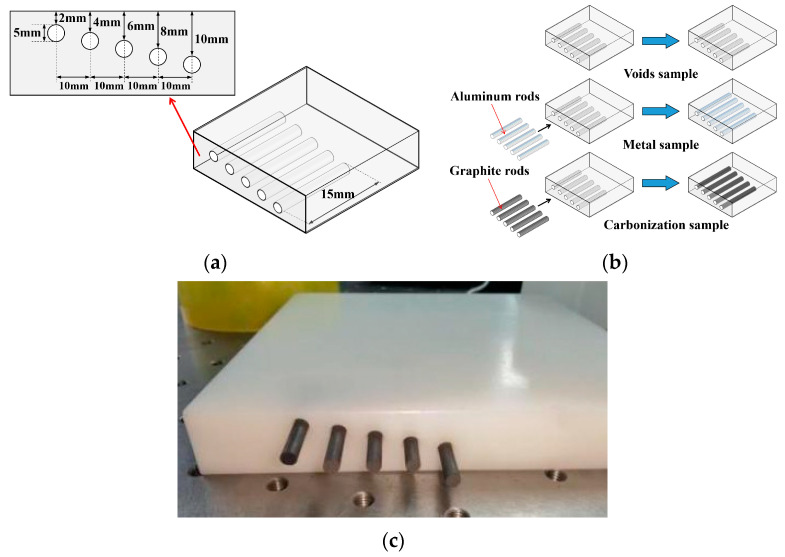
Artificial interface samples for testing the LSTM performance in 3D imaging: (**a**) shape and size; (**b**) three different types of interface; (**c**) picture of the real carbonization sample.

In addition, another PE sample was used in this paper to test the performance of the trained LSTM, and the size and shape of the sample are shown in Figure 4a. The sample was a PE block with five holes, which can simulate voids at different depths in the polymer. As shown in Figure 4b, by inserting aluminum rods and graphite rods in the holes, the metal interfaces and the carbonized interfaces in the polymer can also be simulated.

### 3.2. THz System

**Figure 5:**
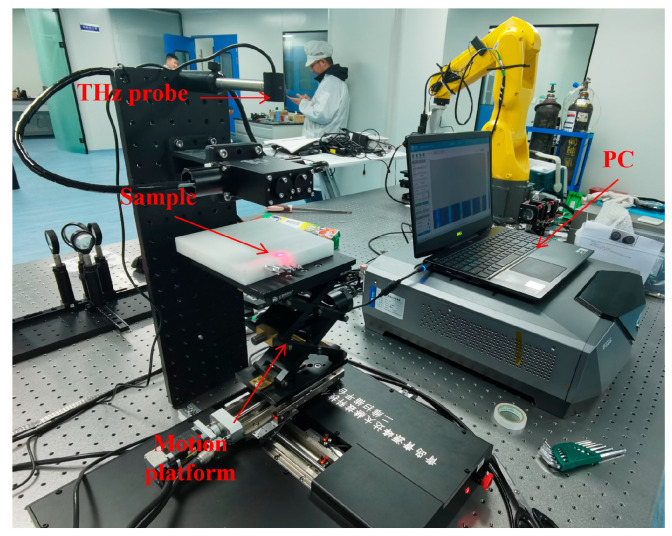
The THz time domain spectroscopy system.

In this paper, QT-TO1000 from Quenda Technology Ltd., China, was used as the THz time domain spectroscopy system, which can emit and receive electromagnetic waves of 0.1–3.5 THz. Its maximum scanning area is up to 100 mm × 100 mm with a step length of 0.3 mm, and its scanning speed is up to 60 pixels per second. As shown in Figure 5, the whole system includes a THz probe, a motion system, and a PC. The THz probe is used to emit and receive the THz waves, and the motion platform is driven by three motors in *X*, *Y*, and *Z* directions, respectively. Both of them are connected to the PC, which controls the motion platform and collects the THz waves’ data to realize the scanning of samples.

## 4. Experiments and Discussion

### 4.1. LSTM Training

**Figure 6:**
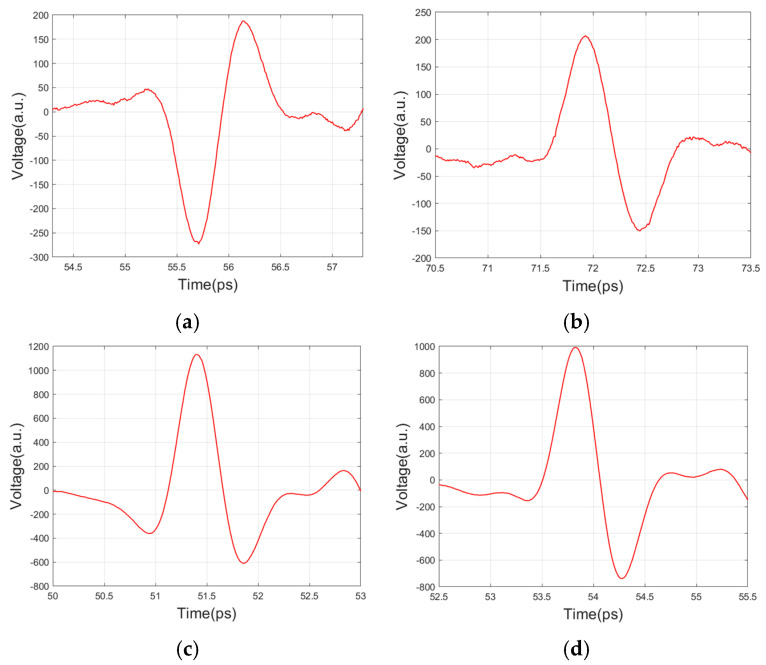
Typical reflected pulse waveforms of artificial interfaces: (**a**) PA interface; (**b**) AP interface; (**c**) PM interface; (**d**) PC interface.

First, waveform data were collected from artificial interface samples for the training of LSTM. The sample was placed on the motion platform. Before collecting data, the height of the platform was adjusted to make sure that the artificial interface was near the focus of the THz beam, in which case the reflected pulse amplitude would reach the maximum. A 50 mm × 50 mm area in the center of three artificial interface samples was scanned, separately, in a step length of 1 mm. Then, the reflected waveform data of the artificial interface samples could be collected. Typical reflected pulse waveforms are shown in Figure 6.

**Figure 7:**
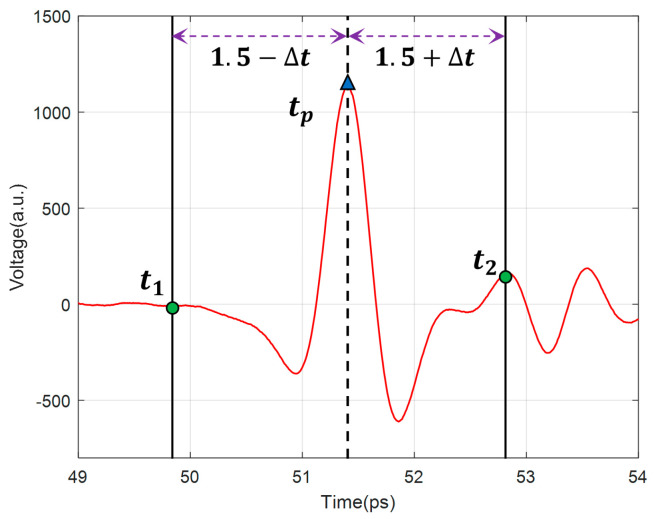
Schematic diagram of pulse data extracting.

After that, the pulse data of the artificial interfaces were extracted from the scanned waveforms. As shown in Figure 7, $t_{1}$ and $t_{2}$ are the start time and end time of the pulse, respectively, and $t_{p}$ is the absolute maximum time (positive or negative peak) of the pulse. The positions of $t_{1}$ and $t_{2}$ can be determined by $t_{p}$, which can be expressed as

(13) $$\left\{\begin{array}{c} t_{1}=t_{p}-1.5+∆t\\ t_{2}=t_{p}+1.5+∆t \end{array}\right.\;\;\;\;\;∆t\in \left[-0.5,\;0.5\right]$$

where ∆t is a random perturbation term, which made the training data more diverse so as to improve the generalization capability of the network.

Then, the pulse data sets of the artificial PA interface $P_{PA}\left(i\right)\;\left(i=1,2,\ldots ,2500\right)$, artificial AP interface $P_{AP}\left(i\right)$, artificial PM interface $P_{PM}\left(i\right)$, and artificial PC interface $P_{PC}\left(i\right)$ can be obtained.

Given that the interfaces inside the actual polymers are usually not exactly at the beam focus, and the amplitude of their reflected pulse will probably decrease. Therefore, the amplitude of the training data should also be randomly reduced to enhance the performance of the trained network. The random reduction process can be expressed as

(14) $$\left\{\begin{array}{c} \begin{array}{c} T_{AP}\left(i\right)=P_{AP}\left(i\right)\cdot f_{AP}\left(i\right)\;\;\;\;\;f_{AP}\left(i\right)\in \left[0.5,\;1\right]\\ T_{PA}\left(i\right)=P_{PA}\left(i\right)\cdot f_{PA}\left(i\right)\;\;\;\;\;f_{PA}\left(i\right)\in \left[0.5,\;1\right] \end{array}\\ \begin{array}{c} T_{PM}\left(i\right)=P_{PM}\left(i\right)\cdot f_{PM}\left(i\right)\;\;f_{PM}\left(i\right)\in \left[0.15,\;1\right]\\ T_{PC}\left(i\right)=P_{PC}\left(i\right)\cdot f_{PC}\left(i\right)\;\;\;\;\;f_{PC}\left(i\right)\in \left[0.15,\;1\right] \end{array} \end{array}\right.$$

where $f_{AP}\left(i\right)$, $f_{PA}\left(i\right)$, $f_{PM}\left(i\right)$, and $f_{PC}\left(i\right)$ are the random reduction factors of the four artificial interfaces, respectively. As a pulse with a too small amplitude is hard to distinguish from the background noise, a threshold value $V_{th}$ should be set. All of the valid pulses must have an absolute maximum larger than $V_{th}$. In this paper, $V_{th}=100$. As shown in Figure 6, different interfaces have different pulse amplitudes. Therefore, the random reduction factors should have different lower limits to ensure all pulses in the training data are valid. Finally, the training set $T\left(i\right)=[T_{AP}\left(i\right),\;T_{PA}\left(i\right)$*,* $T_{PM}\left(i\right)$*,* $T_{PC}\left(i\right){]}^{T}$ can be obtained.

The training set was divided into two parts. One part was used to train the LSTM with 2250 pulse data for each type of interface, another one was as the validation set to test the accuracy of the trained LSTM. The LSTM network was established and was trained by the training set. The input dimension of LSTM was 300, which was the same as the length of pulse data. The LSTM layer was the bi-directional structure with 100 hidden units. The maximum number of iterations was 1200, the learning rate was 0.01, and the gradient threshold was 1.

**Figure 8:**
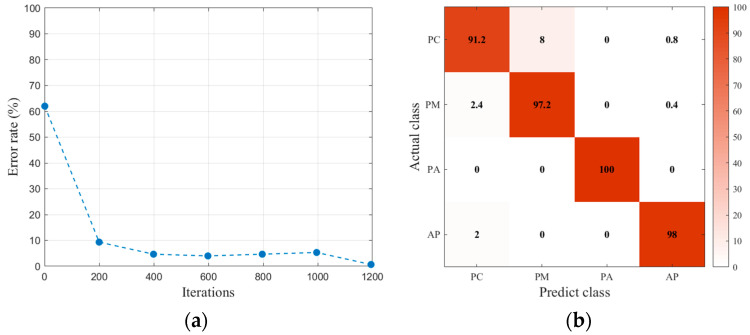
Training results of LSTM: (**a**) training error rate of LSTM; (**b**) classification accuracy of the validation set.

The training results are shown in Figure 8. It can be seen that this LSTM has a good classification performance for the artificial interfaces.

### 4.2. 3D Identification Imaging Test

The holes in the PE block can be imaged in 3D as follows: After the PE block sample was scanned, all the valid pulses in the scanning waveforms data were extracted by the rule of Equation (13) with ∆t=0, and $V_{th}=100$. Each pulse corresponded to a point of a certain interface. According to basic optics laws, its location can be calculated by

(15) $$\left(x,y,z\right)=\left(j\cdot d,\;k\cdot d,-\frac{c_{0}}{2n_{PE}}\cdot t_{p}\right)$$

where $\left(x,y,z\right)$ is the spatial coordinates of the point, j and k are the numbers of scanning steps in the *X* direction and the *Y* direction, respectively, d is the scanning step length, $c_{0}$ is the vacuum light speed, and $n_{PE}$ is the refraction index of PE. Depending on the composition and wave frequency, $n_{PE}$ is in a range of 1.51–1.54 [35]. In this paper, $n_{PE}=1.53$. As the THz probe was above the sample as shown in Figure 5, the *z*-axis coordinate of each point should be negative.

After that, the pulse data are classified by the trained LSTM, and each point will be labeled with a certain interface class. Thus, 3D identification imaging can be realized.

**Figure 9:**
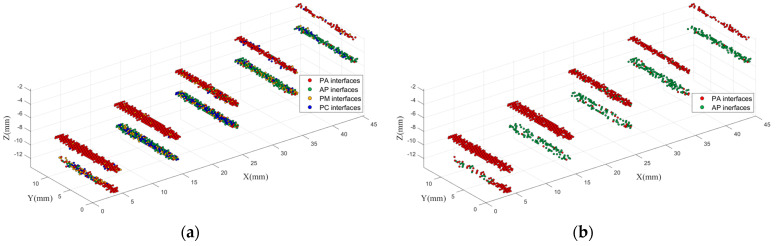

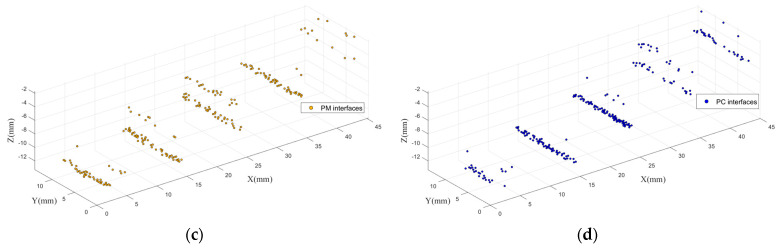
3D identification imaging results of PE block sample of voids. (**a**) Results of all identified points; (**b**) results of points identified as voids; (**c**) results of points identified as metal; (**d**) results of points identified as carbonization.

As the attenuation of propagating in material, deviation from the focus, and the non-flat interface structures, the interfaces inside the PE block sample are more complicated and more similar to an actual situation. Therefore, the PE block sample can be used to test the feasibility of 3D identification imaging with trained LSTM in a relatively actual situation. The 3D identification imaging results of the PE block sample of voids are shown in Figure 9.

As it can be seen, the upper surfaces of the voids are identified better than the lower surfaces. Let I–V represent the five holes at depths from 2 mm to 10 mm, respectively. The number of different points in the result of the PE block sample of voids is listed in Table 1.

From Table 1, the PA interfaces are identified well at all depths. Compared with the PA interfaces, both the correct identification rates and the numbers of points of the AP interfaces are lower. However, for AP interfaces I–IV, the AP points are still the most. For void V, the identification performance of the AP interface is poor. The reason could be that the interface was greatly out of focus, which caused strong attenuation of the THz waves. It also can be seen that the number of all points varied greatly with depth. Therefore, the depth could greatly influence the imaging performance of the voids.

**Figure 10:**
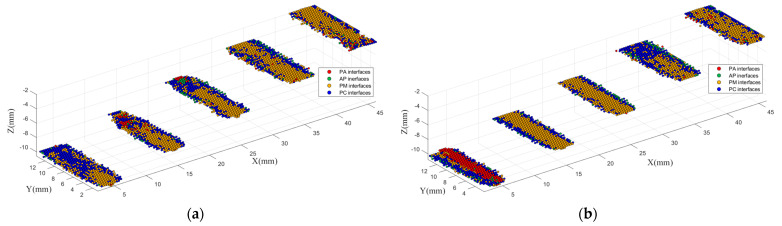
3D identification imaging results of PE block sample of metal and carbonization: (**a**) PE block sample of metal; (**b**) PE block sample of carbonization.

The PE block samples of the metal and carbonization were also imaged in the same way, respectively. The results are shown in Figure 10 and Table 2 and Table 3.

**Figure 11:**
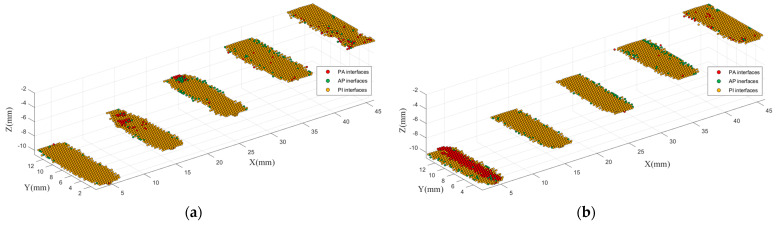
3D identification imaging results with three classes of interface: (**a**) PE block sample of metal; (**b**) PE block sample of carbonization.

As it can be seen, compared with voids, the depth does not affect the imaging performance much for the PM interfaces and the PC interfaces. On the other hand, the PM interfaces and the PC interfaces tend to be confused, which is different from the results of the training. In particular, the PC interfaces are easily misidentified as the PM interfaces. However, both the PM and the PC interfaces are less likely to be identified as the PA interfaces or the AP interfaces. Therefore, the PM interfaces and the PC interfaces can be combined as “polymer−impurity” (PI) interfaces to solve the confusion problem. As both the metal and carbonization are solid impurities, this method is still meaningful in practical application. The imaging results with three classes are shown in Figure 11. After combination, the average correct identification rates for PE block samples of metal and carbonization are up to 90.95% and 87.28%, respectively.

## 5. Conclusions

In this paper, a method based on the THz waves and LSTM classification network was proposed to identify the interface structures inside polymers. LSTM learns from the artificial interface samples firstly, then the trained LSTM is used to identify the internal interfaces from other samples to achieve 3D identification imaging. The experiment results showed that the proposed method could identify the voids interfaces well, while the metal and carbonization interfaces tended to be confused. By combining the metal and the carbonization interfaces as the impurity interface, the identification results were satisfactory.

## Figures and Tables

**Table 1:** Number of different points in the imaging results of the PE block sample of the voids.

Actual Interface	Number of PA Points	Number of AP Points	Number of PM Points	Number of PC Points	Correct Identification Rate
PA interface I	57	0	5	3	87.69%
AP interface I	5	89	8	26	69.53%
PA interface II	118	3	2	15	85.51%
AP interface II	12	102	46	22	56.04%
PA interface III	161	13	21	6	80.10%
AP interface III	15	75	38	69	38.07%
PA interface IV	244	0	9	1	96.06%
AP interface IV	6	85	48	60	42.71%
PA interface V	230	0	6	5	95.44%
AP interface V	66	17	37	21	12.06%

**Table 2:** Number of different points in the imaging results of the PE block sample of metal.

Actual Interface	Number of PA Points	Number of AP Points	Number of PM Points	Number of PC Points	Correct Identification Rate
PM interface I	33	16	423	163	66.61%
PM interface II	11	41	459	262	59.38%
PM interface III	48	50	415	304	50.80%
PM interface IV	80	19	478	237	58.72%
PM interface V	51	12	533	357	55.93%

**Table 3:** Number of different points in the imaging results of the PE block samples of carbonization.

Actual Interface	Number of PA Points	Number of AP Points	Number of PM Points	Number of PC Points	Correct Identification Rate
PC interface I	23	15	353	158	28.78%
PC interface II	5	58	240	293	49.16%
PC interface III	16	28	416	115	20.00%
PC interface IV	55	21	533	154	20.18%
PC interface V	231	38	348	328	34.71%

## Data Availability

Data presented in this study are available on request from the first author.
